# Supplementary material for: Circadian regulation of night feeding and daytime detoxification in a formidable Asian pest Spodoptera litura
Source: Commun Biol. 2021 Mar 5;4:286. doi: 10.1038/s42003-021-01816-9 (PMC7935888; doi:10.1038/s42003-021-01816-9)
Supplement: Supplementary file 2 — Supplementary Information [file 42003_2021_1816_MOESM2_ESM.pdf]

Circadian regulation of night feeding and daytime detoxification in a formidable Asian pest *Spodoptera litura*

Jiwei Zhang[1,2], Shenglong Li[1], Wanshun Li[1,2], Zhiwei Chen[1], Huizhen Guo[1,2], Jianqiu Liu[2], Yajing Xu[1,2], Yingdan Xiao[1,2], Liying Zhang[1,2], Kallare P. Arunkumar[3], Guy Smagghe[4,5], Qingyou Xia[1,2], Marian R. Goldsmith[6*], Makio Takeda[7*], Kazuei Mita[1,2*]

[1]State Key Laboratory of Silkworm Genome Biology, Southwest University, Chongqing, 400716, China.

[2]Biological Science Research Center, Southwest University, Chongqing, 400715, China.

[3]Central Muga Eri Research and Training Institute, (CMER&TI), Central Silk Board, Lahdoigarh, Jorhat 785700, India.

[4]College of Plant Protection and Academy of Agricultural Sciences, Southwest University, Chongqing 400716, China.

[5]Department of Plants and Crops, Laboratory of Agrozoology and International Joint China-Belgium Laboratory on Sustainable Control of Crop Pests, Ghent University, 9000 Ghent, Belgium.

[6]University of Rhode Island, Kingston, RI, 02881 USA.

[7]Graduate School of Agricultural Science, Kobe University, Kobe 657-8501, Japan.

*Corresponding authors:

Kazuei Mita

State Key Laboratory of Silkworm Genome Biology, Southwest University, Chongqing 400716, China. E-mail: mitakazuei@gmail.com.

Marian R. Goldsmith

Department of Biological Sciences, University of Rhode Island, Kingston, RI, 02881 USA. E-mail: mki101@uri.edu.

Makio Takeda

Graduate School of Agricultural Science, Kobe University, Kobe 657-8501, Japan. E-mail: mtakeda@kobe-u.ac.jp.

Supplementary Information

Supplementary Table 1. Annotation of 9 core circadian clock genes in *S. litura*

Gene name	Gene ID	Chr#	Chromosomal position		Dir	CDS(bp)	aa length	# of Exons
SlituPer	SWUSl0003910	Z	13178586	13208342	-	3723	1240	30
SlituClk	SWUSl0003660	Z	12487604	12503915	-	1860	619	13
SlituBmal1	SWU0005160	Z	18763637	18791067	+	2109	702	12
SlituCwo	SWUSl0139980	Scaffold 476	128179	134697	-	1320	439	9
SlituTim	SWUSl0067960	15	6136748	6144902	+	3831	1276	16
SlituCry1	SWUSl0127090	29	2453421	2463313	-	1647	548	13
SlituCry2	SWUSl0014350	2	8443820	8452904	+	2445	814	10
SlituVri	SWUSl0020450	4	3453325	3465178	+	1254	417	3
SlituPdpε1	SWUSl0005220	Z	18891493	18899973	+	813	270	6

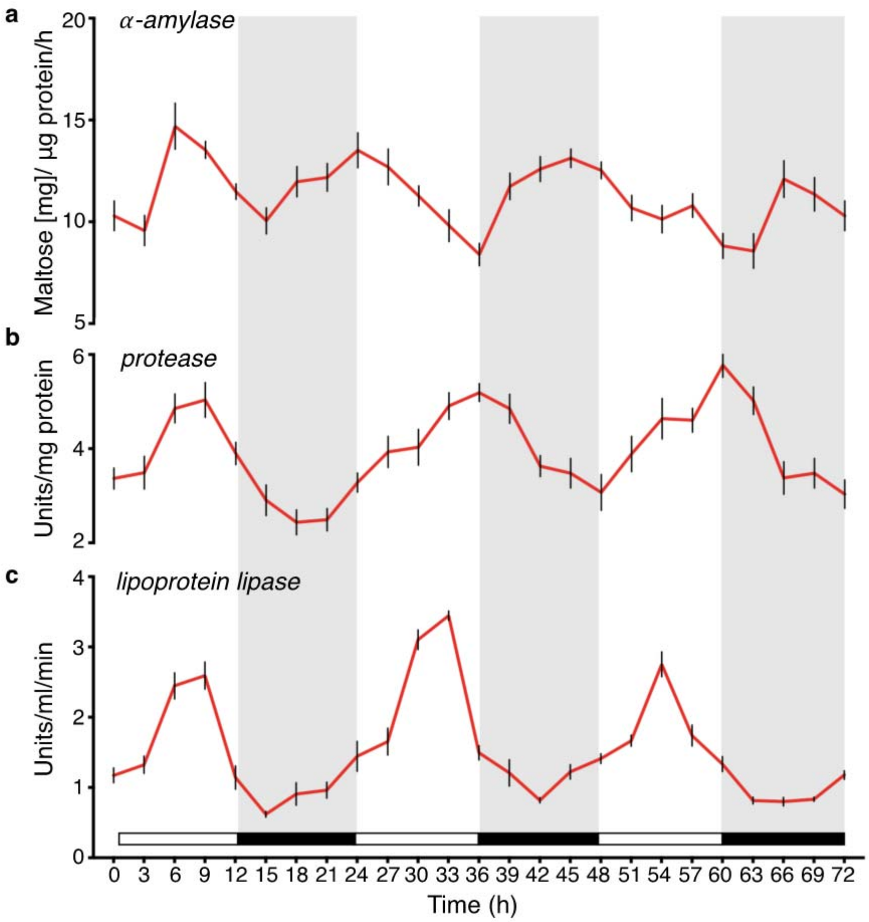

Supplementary Fig. 1 Daily rhythms of digestive enzyme activity in the midgut lumen during 6LD1 to 6LD3. (**a**) α-amylase activity; (**b**) total protease activity; (**c**) lipoprotein lipase activity. Three groups with 3 individuals each were used for midgut extraction. The photophase is marked by white rectangles, and the scotophase by black rectangles and black shading. Means ± SEM are plotted.

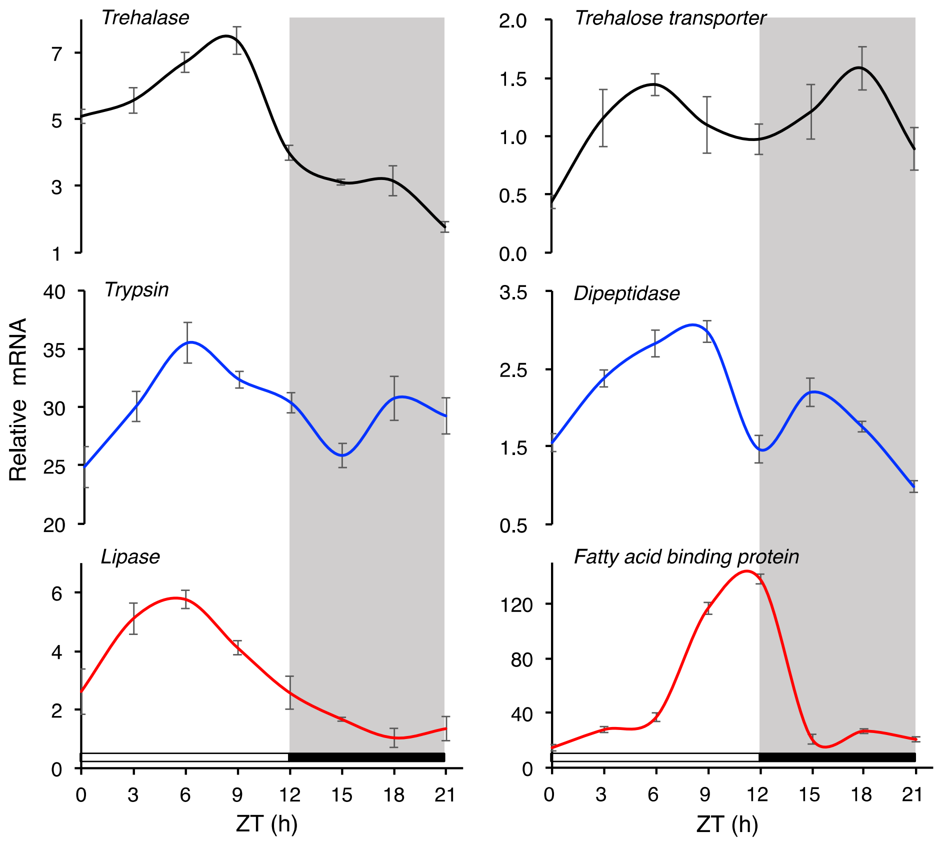

Supplementary Fig. 2 Transcriptional analysis of digestion-related genes involved in carbohydrate, lipid and protein metabolism in the midgut by qPCR. Each experiment was performed with midguts of 3 6LD2 larvae and repeated independently three times. White and black shading indicates the photophase and scotophase. Error bars represent ± SEM.

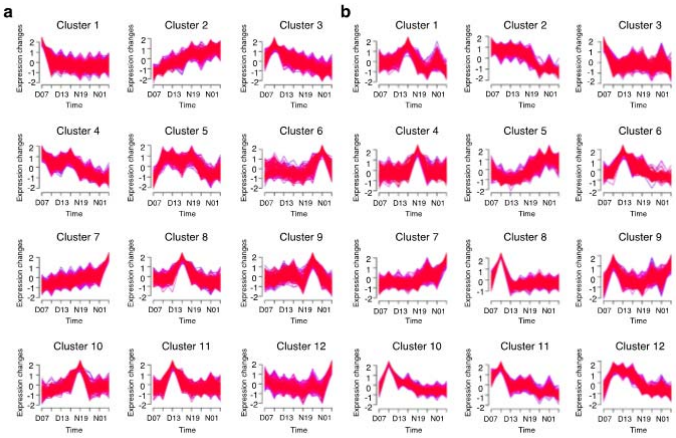

Supplementary Fig. 3 Cluster analyses of transcripts with differential expression patterns in the midgut and fat body using the Mfuzz method. (**a**) midgut; (**b**) fat body. The expression values were calculated with RNA-seq data and were processed with the method of homogenization by log10. Midguts and fat bodies from 6LD1 larvae were collected for RNA sequencing at 3 h intervals in 24 h.

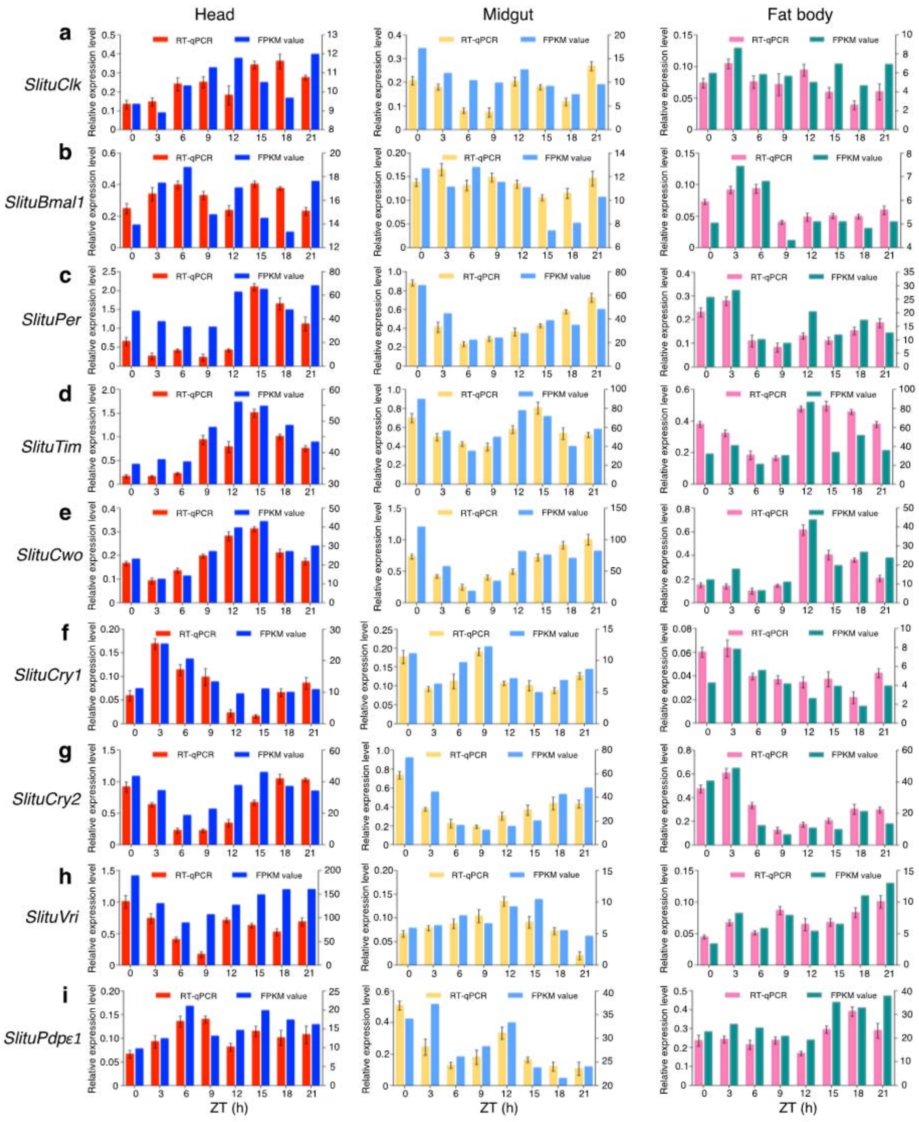

Supplementary Fig. 4 Daily expression analyses of core circadian genes in head, midgut, and fat body of *S. litura* larvae. The 24 h expression patterns are presented in each graph as *SlituClk* (**a**), *SlituBmal1* (**b**), *SlituPer* (**c**), *SlituTim* (**d**), *SlituCwo* (**e**), *SlituCry1* (**f**), *SlituCry2* (**g**), *SlituVri* (**h**) and *SlituPdpε1* (**i**). The left ordinate is the relative expression with real-time PCR detection (±SEM) and the right ordinate is the FPKM value based on the RNA-seq data. Red and dark blue represent RT-qPCR and FPKM values in the head. Yellow and light blue represent RT-qPCR and FPKM values in the midgut. Pink and dark green represent RT-qPCR and FPKM values in the fat body. Three groups of 3 6LD2 individuals were used for each RT-qPCR test. Each value represents the average of three experiments and means ± SEM are plotted. Timing of photophase and scotophase correspond to Supplementary Fig. 2.

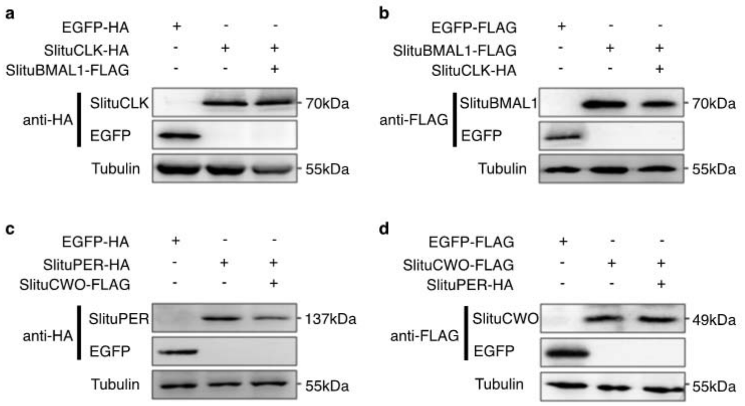

Supplementary Fig. 5 Overexpression of circadian core genes in Spli221 cells determined by Western blotting. (**a**) CLK, (**b**) BMAL1 (**c**) PER, and (**d**) CWO. SlituCLK and SlituPER were designed to be tagged with HA and SlituBMAL1 and SlituCWO with FLAG. The over-expressed EGFP was set as corresponding control.

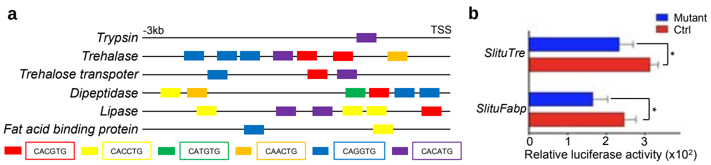

Supplementary Fig. 6 E-box annotation and location in the promoters of digestion-related genes and E-box mutant analysis for *SlituTre* (*Trehalase*) and *SlituFabp* (*Fat acid binding protein*). **a** Distributions of canonical (CACGTG) and non-canonical (CANNTG) E-box sequences in 5' regulatory regions of selected digestion-related genes. **b** The relative luciferase activity of mutated (TGTACT, blue) and normal (CACGTG, red) E-box sequences of *SlituTre* and *SlituFabp* induced by co-transfection with over-expression *SlituClk*. The relative luciferase activities represent the relative transcription activities of *SlituTre* and *SlituFabp* and the values are given as mean ± SEM of three repeated experiments. The statistically significant difference was set at * P value < 0.05, **P value < 0.01 and ***P value < 0.001.
