# Supplementary material for: Circadian regulation of night feeding and daytime detoxification in a formidable Asian pest Spodoptera litura
Source: Commun Biol. 2021 Mar 5;4:286. doi: 10.1038/s42003-021-01816-9 (PMC7935888; doi:10.1038/s42003-021-01816-9)
Supplement: Supplementary file 3 — Description of Additional Supplementary Files [file 42003_2021_1816_MOESM3_ESM.pdf]

Description of Additional Supplementary Files

File name: Supplementary Data 1

Description: Primers and siRNA sequences used for RT-qPCR and other experiments.

File name: Supplementary Data 2

Description: RT-qPCR raw data of Fig. 3.

File name: Supplementary Data 3

Description: List of detoxification genes and their E-Box locations for Fig. 5.

File name: Supplementary Data 4

Description: The source data for the main figures (Fig. 1, 2, 4, 6 and 7).
