# Supplementary material for: Circadian regulation of night feeding and daytime detoxification in a formidable Asian pest Spodoptera litura
Source: Commun Biol. 2021 Mar 5;4:286. doi: 10.1038/s42003-021-01816-9 (PMC7935888; doi:10.1038/s42003-021-01816-9)
Supplement: Supplementary file 8 — Reporting Summary [file 42003_2021_1816_MOESM8_ESM.pdf]

nature research

Corresponding author(s): Kazuei Mita

Last updated by author(s): Jan 30, 2021

Reporting Summary

Nature Research wishes to improve the reproducibility of the work that we publish. This form provides structure for consistency and transparency in reporting. For further information on Nature Research policies, see our Editorial Policies and the Editorial Policy Checklist.

Statistics

For all statistical analyses, confirm that the following items are present in the figure legend, table legend, main text, or Methods section.

n/a	Confirmed	
☐	☒	The exact sample size (*n*) for each experimental group/condition, given as a discrete number and unit of measurement
☐	☒	A statement on whether measurements were taken from distinct samples or whether the same sample was measured repeatedly
☐	☒	The statistical test(s) used AND whether they are one- or two-sided<br>*Only common tests should be described solely by name; describe more complex techniques in the Methods section.*
☒	☐	A description of all covariates tested
☐	☒	A description of any assumptions or corrections, such as tests of normality and adjustment for multiple comparisons
☐	☒	A full description of the statistical parameters including central tendency (e.g. means) or other basic estimates (e.g. regression coefficient) AND variation (e.g. standard deviation) or associated estimates of uncertainty (e.g. confidence intervals)
☐	☒	For null hypothesis testing, the test statistic (e.g. *F*, *t*, *r*) with confidence intervals, effect sizes, degrees of freedom and *P* value noted<br>*Give P values as exact values whenever suitable.*
☒	☐	For Bayesian analysis, information on the choice of priors and Markov chain Monte Carlo settings
☒	☐	For hierarchical and complex designs, identification of the appropriate level for tests and full reporting of outcomes
☒	☐	Estimates of effect sizes (e.g. Cohen's *d*, Pearson's *r*), indicating how they were calculated

Our web collection on statistics for biologists contains articles on many of the points above.

Software and code

Policy information about availability of computer code

Data collection	Spodoptera litura genome information in NCBI BioProject accession: PRJNA344815
Data analysis	clustering analysis using Mfuzz software with FCM parameters of c = 12, m = 1.25 (http://mfuzz.sysbiolab.eu), which is a package based on R with a soft clustering algorithm. siRNA and primer designs were performed by GENETYX-MAC version 16.0.7

For manuscripts utilizing custom algorithms or software that are central to the research but not yet described in published literature, software must be made available to editors and reviewers. We strongly encourage code deposition in a community repository (e.g. GitHub). See the Nature Research guidelines for submitting code & software for further information.

Data

Policy information about availability of data

All manuscripts must include a data availability statement. This statement should provide the following information, where applicable:

- Accession codes, unique identifiers, or web links for publicly available datasets
- A list of figures that have associated raw data
- A description of any restrictions on data availability

RNA-Seq data for transcriptome analysis have been submitted to the NCBI Short Read Archive under accession number PRJNA511360 and the annotated sequences have been submitted to GenBank under BioProject accession PRJNA344815. All other data generated or analysed during this study are included in this published article and its Supplementary Information and Supplementary Data files. All relevant data are available from corresponding author upon request (to mitakazuei@gmail.com).

Field-specific reporting

Please select the one below that is the best fit for your research. If you are not sure, read the appropriate sections before making your selection.

☒ Life sciences ☐ Behavioural & social sciences ☐ Ecological, evolutionary & environmental sciences

For a reference copy of the document with all sections, see nature.com/documents/nr-reporting-summary-flat.pdf

Life sciences study design

All studies must disclose on these points even when the disclosure is negative.

Sample size	For behavioural observation experiments, sample size is written in Materials and Methods. For transcriptome analysis, we described how many RNAs are used in Materials and Methods.
Data exclusions	No data exclusion in this work.
Replication	In Figure legends, each experiment has been replicated three times.
Randomization	All samples were randomly selected.
Blinding	Investigators were blinded to group allocation during data collection.

Reporting for specific materials, systems and methods

We require information from authors about some types of materials, experimental systems and methods used in many studies. Here, indicate whether each material, system or method listed is relevant to your study. If you are not sure if a list item applies to your research, read the appropriate section before selecting a response.

Materials & experimental systems

n/a	Involved in the study	
☒	☐	Antibodies
☐	☒	Eukaryotic cell lines
☒	☐	Palaeontology and archaeology
☐	☒	Animals and other organisms
☒	☐	Human research participants
☒	☐	Clinical data
☒	☐	Dual use research of concern

Methods

n/a	Involved in the study	
☒	☐	ChIP-seq
☒	☐	Flow cytometry
☒	☐	MRI-based neuroimaging

Eukaryotic cell lines

Policy information about cell lines

Cell line source(s)	Spodoptera litura embryonic cell line Spli-221
Authentication	Our laboratory established this cell line and maintained
Mycoplasma contamination	The cell line was not tested for mycoplasma contamination.
Commonly misidentified lines (See ICLAC register)	No misidentified line

Animals and other organisms

Policy information about studies involving animals; ARRIVE guidelines recommended for reporting animal research

Laboratory animals	Spodoptera litura Ishihara strain, Sex: mixed, stage:larval stage
Wild animals	No
Field-collected samples	No
Ethics oversight	For Spodoptera litura, no regulation exists.

Note that full information on the approval of the study protocol must also be provided in the manuscript.
